# Supplementary material for: Application of qualifying variants for genomic analysis
Source: Bioinformatics. 2026 Jan 22;42(2):btaf676. doi: 10.1093/bioinformatics/btaf676 (PMC12926777; doi:10.1093/bioinformatics/btaf676)
Supplement: btaf676_Supplementary_Data [file btaf676_supplementary_data.pdf]

Supplemental: Application of qualifying variants for genomic analysis

Dylan Lawless*[1], Ali Saadat[2], Mariam Ait Oumelloul[2], Simon Boutry[2], Veronika Stadler[1], Sabine Österle[3], Jan Armida[3], David Haerry[4], D. Sean Froese[5], Luregn J. Schlapbach[1], and Jacques Fellay[2]

[1]Department of Intensive Care and Neonatology, University Children's Hospital Zürich, University of Zürich, Switzerland.
[2]Global Health Institute, School of Life Sciences, École Polytechnique Fédérale de Lausanne, Switzerland.
[3]SPHN Data Coordination Center, SIB Swiss Institute of Bioinformatics, Basel, Switzerland.
[4]Positive Council, Zürich, Switzerland.
[5]Division of Metabolism and Children's Research Center, University Children's Hospital Zürich, University of Zurich, Zurich, Switzerland.

December 16, 2025

*Addresses for correspondence: Dylan.Lawless@kispi.uzh.ch

1 Supplemental

Application of qualifying variants for genomic analysis.

1.1 Validation study figures

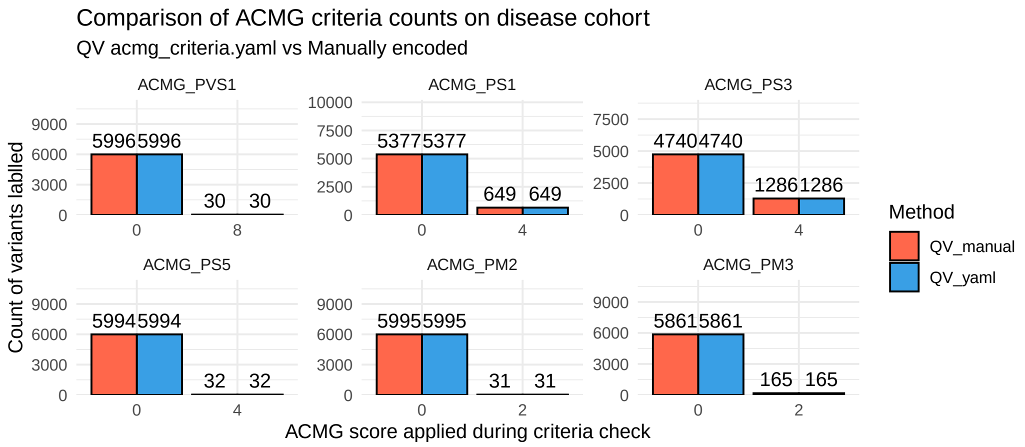

Figure S1: Validation case study of a rare disease cohort of 940 WES individuals using an ACMG criteria subset, demonstrating a 100% match between manually encoded and standalone YAML-based QV for assigning pathogenicity scores.

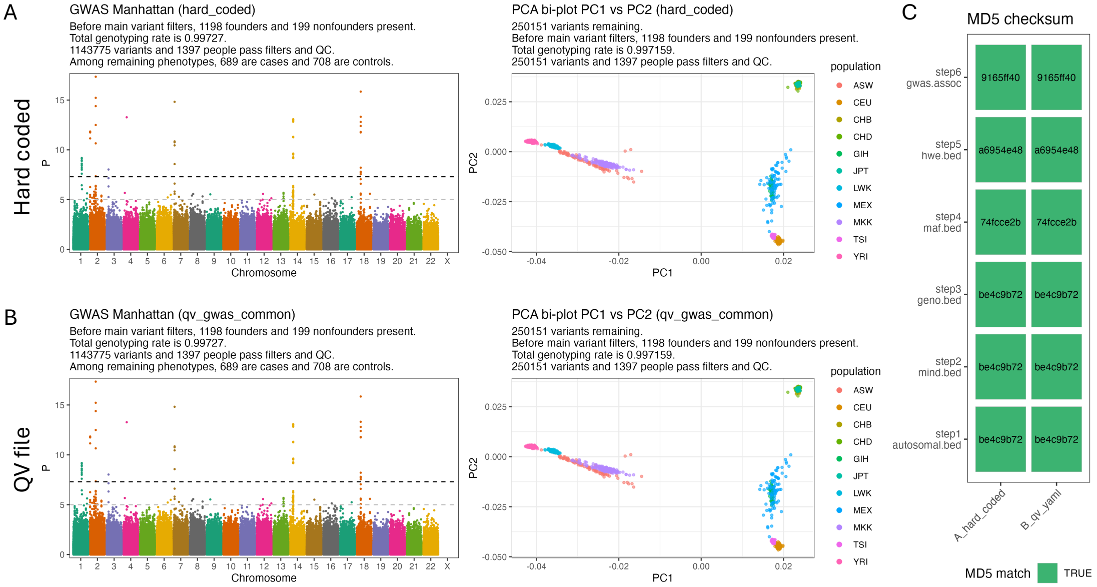

Figure S2: Validation in GWAS using QV parameterisation. (A) GWAS of simulated binary phenotypes in HapMap3 Phase 3 (R3) using a traditional variable embedded pipeline. Shown are the Manhattan plot of logistic regression results (left) and correction for population structure with principal component analysis (PC1 vs PC2, right). (B) Identical GWAS using a QV YAML configuration file. The Manhattan and PCA results are indistinguishable from panel A. (C) Verification of reproducibility. MD5 checksums of the main PLINK outputs are identical between panels A and B. The steps included processing of autosomal biallelic SNPs, sample call rate, variant call rate, minor allele frequency, Hardy–Weinberg equilibrium, and association results. The QV file encoded these thresholds (sample call rate ≥95%, variant call rate ≥95%, MAF ≥1%, HWE p ≥1e-6, autosomal biallelic SNPs only) together with covariates (sex and PC1-PC10) and logistic regression settings. This confirms that a shareable QV file reproduces hard-coded pipelines exactly while improving transparency and reusability.

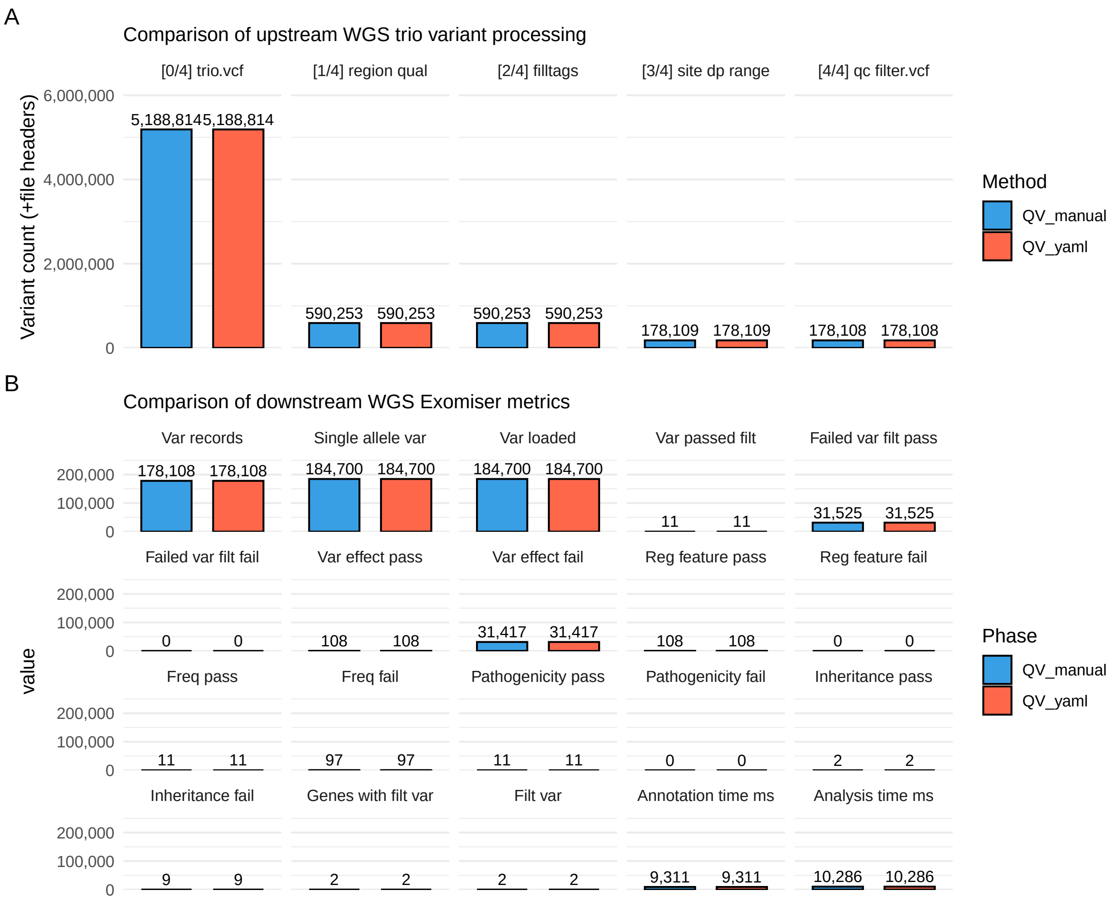

C

Comparison of top two Exomiser variants

phase	MOI	Effect	HGVS	Genotype
QV_manual	AD	frameshift	RYR2:ENST00000366574.7:c.6493dup:p.(Leu2165Profs*44)	0/1:./.:./.
QV_yaml	AD	frameshift	RYR2:ENST00000366574.7:c.6493dup:p.(Leu2165Profs*44)	0/1:./.:./.
QV_manual	AR	missense	TTN:ENST00000589042.5:c.36982A>G:p.(Thr12328Ala)	1|1:0/1:0/1
QV_yaml	AR	missense	TTN:ENST00000589042.5:c.36982A>G:p.(Thr12328Ala)	1|1:0/1:0/1

Figure S3: Validation of the trio Exomiser pipeline using QV parameterisation. **(A)** summarises upstream processing counts by file, **(B)** compares downstream Exomiser metrics, and **(C)** shows the key variant fields for the two variants identified. Variant counts in all panels confirm that intermediate files and final outputs are identical between configurations. The five preprocessing stages shown in (A) are: (0) input trio VCF, (1) gene panel region and quality filtering, (2) tag annotation, (3) site-level depth range filtering, and (4) final QC-filtered VCF. MOI, mode of inheritance; HGVS, Human Genome Variation Society nomenclature.

1.2 Computational benchmark

Runtime performance was equivalent between traditional and QV-based pipelines, as both read identical parameters from different sources. In the WGS trio validation study, pre-processing steps including filtering, Quality Control (QC), and gene panel selection completed in 16–17 seconds, with a median difference of ~0.5 seconds favouring the QV YAML pipeline (**Figure** S4). An incidental one-off 5 second delay arose from Singularity initialisation for the `yq` utility (step 0), a system-specific effect on our HPC and unrelated to the framework itself.

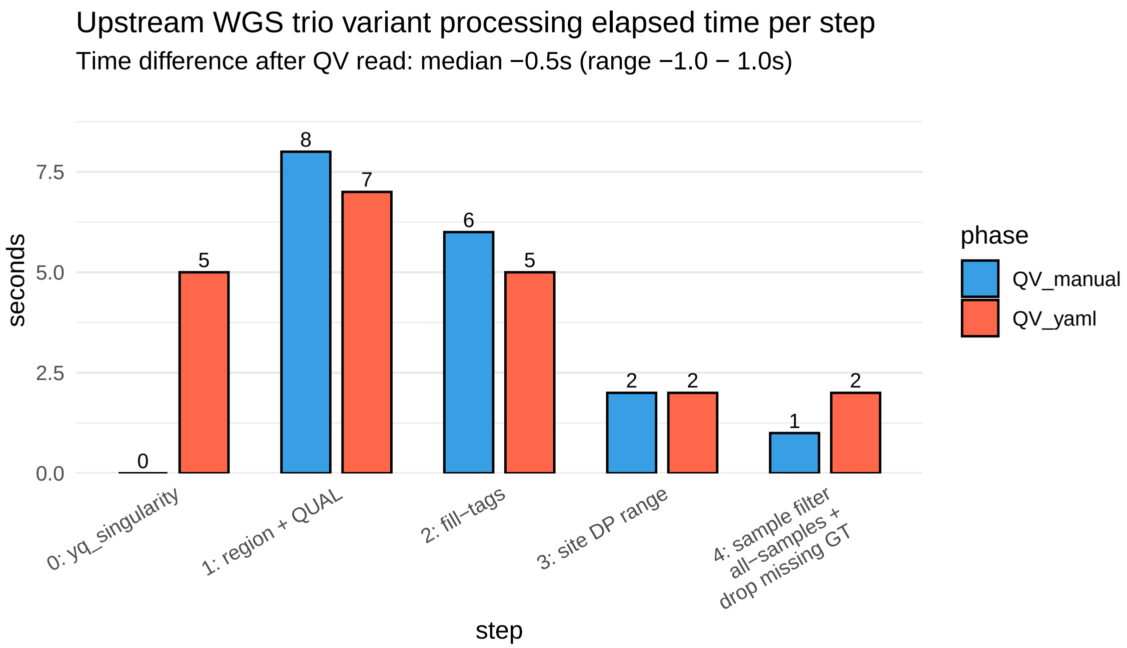

Figure S4: Benchmark of upstream preprocessing times in the WGS trio pipeline comparing QV-based and traditional (manually parameterised) configurations. Stepwise elapsed times were nearly identical across both methods (median difference ~0.5 s), with a fixed 5 s overhead from optional Singularity initialisation of `yq` in the QV pipeline. The four preprocessing steps correspond to: (1) gene panel region and quality filtering of the trio VCF, (2) annotation of variant tags, (3) site-level depth range filtering, and (4) per-sample genotype filtering and exclusion of missing genotypes. All steps used `BCFtools` on VCF preprocessing, as illustrated in **Figure** S3 **(A)**.

1.3 How to build a QV file

We recommend YAML or JSON for portability and adoption. You can build a QV in three ways:

Option 1: use the HTML QV builder (Zenodo)

1. Open the HTML builder from the Zenodo repository.
2. Enter simple `key=value` statements in the left pane.
3. Copy or download the generated YAML.

Example input lines:

```
meta qv_set_id="qv_gwas_common_v1_20250827"
meta version="1.0.0"
meta title="GWAS common QC"
meta authors=Alice,Bob
meta tags=GWAS,QC,PCA
filter maf_minimum field=MAF operator=">=" value=0.01 desc="Minimum MAF"
filter hwe field=HWE_P operator=">=" value=1e-6 logic=keep_if
filter region_include desc="include panel" field=OVERLAP(targets.exome.bed)
    >>>> operator=">=" value=1 logic=keep_if
criteria disease_panel logic=and desc="HIGH impact within panel"
criteria disease_panel field=IMPACT operator="==" value=HIGH
criteria disease_panel field=OVERLAP(targets.exome.bed) operator=">=" value=1
meta description_patient=
    >>>> "There is a strong family history of early heart attacks."
meta description_ppie=
    >>>> "The PPIE group reviewed and approved the criteria on 2025-08-15."
```

Option 2: write YAML by hand

Minimal pattern:

```
meta:
  qv_set_id: qv_disease_panel_v1_20250828
  version: 1.0.0
  title: Disease panel filter
filters:
  region_include:
    description: Restrict to curated disease gene panel
    logic: keep_if
```

```
    field: OVERLAP(targets.disease_panel.bed)
    operator: ">="
    value: 1
criteria:
  pathogenic:
    description: Variant classified as pathogenic or likely pathogenic
    logic: and
    conditions:
      - group: any_of:start
      - { field: CLASS, operator: "==", value: P }
      - { field: CLASS, operator: "==", value: LP }
      - group: any_of:end
notes:
  - Gene panel file defines the target regions
```

Option 3: write JSON

JSON equivalent of the minimal example:

```
{
  "meta": {
    "qv_set_id": "qv_disease_panel_v1_20250828",
    "version": "1.0.0",
    "title": "Disease panel filter"
  },
  "filters": {
    "region_include": {
      "description": "Restrict to curated disease gene panel",
      "logic": "keep_if",
      "field": "OVERLAP(targets.disease_panel.bed)",
      "operator": ">=",
      "value": 1
    }
  },
  "criteria": {
    "pathogenic": {
      "description": "Variant classified as pathogenic or likely pathogenic",
      "logic": "and",
```

```
      "conditions": [
        { "group": "any_of:start" },
        { "field": "CLASS", "operator": "==", "value": "P" },
        { "field": "CLASS", "operator": "==", "value": "LP" },
        { "group": "any_of:end" }
      ]
    }
  },
  "notes": [
    "Gene panel file defines the target regions"
  ]
}
```

Checksum and register

Record the checksum and register the release:

```
sha256sum qv/examples/qv_disease_panel_v1_20250828.yaml
```

```
# qv/registry/releases.csv
qv_set_id, version, checksum, file, date
qv_disease_panel_v1_20250828,1.0.0, ef6cf810b994...,
    > qv_disease_panel_v1_20250828.yaml, 2025-08-28
```

Versioning and IDs

Use a stable `qv_set_id` plus semantic version. Update the version on any change that affects selection or interpretation. Keep one file per release and never mutate published files.

Use in a workflow

Point your pipeline to the QV file:

```
# workflows/.../config.yaml
qv_file: ".../qv/registry/qv_disease_panel_v1_20250828.yaml"
```

It can be read programmatically at runtime, for example using `yq` in shell-based workflows or `yaml::read_yaml()` in R, providing the same parameters that would otherwise be embedded within pipeline configurations.

Acronyms

ACMG American College of Medical Genetics and Genomics

GWAS Genome-Wide Association Study

QC Quality Control

QV Qualifying Variant
